# Assessment of the metal pollution in surface sediments of coastal Tasaul Lake (Romania)

**DOI:** 10.1007/s10661-020-08698-0

**Published:** 2020-11-05

**Authors:** Dan Vasiliu, Andra Bucse, Naliana Lupascu, Bogdan Ispas, Catalin Gheablau, Ion Stanescu

**Affiliations:** 1 National Institute for Research and Development on Marine Geology and Geoecology (GeoEcoMar), 23-25 Dimitrie Onciul, 024053 Bucharest, Romania; 2 Chemical and Biochemical Engineering Department, University Politehnica of Bucharest, 1-3 Gheorghe Polizu, 011061 Bucharest, Romania

**Keywords:** Metals, Surface sediments, Tasaul Lake, Pollution indices, Coastal lakes

## Abstract

Forty-three surface sediment samples were collected in September 2019 from Tasaul Lake (Black Sea coast, Romania) to examine the metal distribution patterns, assess the level of metal contamination, and identify the pollutant sources. The determined mean metal concentrations were as follows: Al 49,772 mg/kg, Zn 84.40 mg/kg, Cr 83.70 mg/kg, V 76.45 mg/kg, Ni 42.53 mg/kg, Cu 34.27 mg/kg, Pb 26.30 mg/kg, As 12.49 mg/kg, and Hg 0.06 mg/kg. The metals in the surface sediments of Tasaul Lake displayed moderate spatial variation, with higher metal concentrations mainly occurring in the south and southeast (As, Pb, and Hg), southwest (Cu and Zn), and west of the lake (Cr, Ni, and V). Heavy metal contamination in sediments is assessed using pollution indices such as enrichment factor, contamination factor, and pollution load index. The highest CFs and EFs were determined for As (moderate to high pollution), followed by Pb (low to moderate pollution). The Cu, Zn, and Hg pollution indices showed values corresponding to low pollution levels, while Ni, Cr, and V presented the lowest indices, suggesting unpolluted sediments. Multivariate statistical analyses were performed to identify the origin of the analyzed heavy metals. Cr was predominantly sourced from lithogenic components, Ni and V originated from both natural and anthropogenic sources, and As, Cu, Zn, Pb, and Hg showed mainly anthropogenic sources such as agricultural runoff, domestic and industrial wastewater discharges, and quarrying activities.

## Introduction

Generally, coastal lakes are threatened ecosystems due to the numerous human activities carried out within their watersheds, resulting in multiple pressures (eutrophication, pollution, overfishing, etc.) that negatively impact ecosystem components and human health. Heavy metal pollution is one of the major pressures impacting lacustrine ecosystems owing to the toxicity, abundance, and persistence of heavy metals in the environment and their subsequent accumulation in the environment and organisms (Rippey et al. 2008; Atici et al. 2008; Varol 2011). Metals enter lakes from different sources, such as rock weathering, wind-borne soil particles, disposal of liquid effluents, terrestrial runoff carrying numerous chemicals resulting from urban, industrial, and agricultural activities, and atmospheric deposition (Jiang et al. 2012) and ultimately are deposited in sediments (Bing et al. 2011). Lake sediments act as either a permanent or temporary sink of metals; thus, their level of contamination provides an overall picture of health status of the lake ecosystem.

There are many coastal lakes along the Romanian Black Sea littoral region, some of them (those located in the south) strongly impacted by human activities (tourism, agriculture, industry, urban extension, etc.). The second largest lake in the Romanian littoral region is Tasaul Lake, located in the central part of the Romanian coast, south of the Danube Delta Biosphere Reserve. Its importance at regional scale results from the ecosystem services offered, particularly provisioning (fishery and water for agriculture purposes) and cultural (recreational and aesthetics).

Although its catchment area is not very densely populated, the human-induced pressures are quite significant, resulting mainly from agriculture, mining activities, and the petrochemical industry (Vasiliu et al. 2007). Studies on the impact of the anthopogenic pressures on Tasaul Lake ecosystem and the services provided are scarce and not readily accessible. The most recent studies were carried out within the framework of the project *Assessment of anthropogenic impacts on Tasaul Lake, Romania and ecosystem rehabilitation* (Swiss-Romanian cooperative program on “Environmental Science and Technology in Romania-ESTROM”) from 2005 to 2008. They were mainly focused on fishery, eutrophication, primary productivity, etc. (Alexandrov et al. 2008; Cernisencu et al. 2007; Vasiliu et al. 2007; Rosioru et al. 2008), and to a lesser extent on the pollution assessment (Oros 2007; Bloesch and Alexandrov 2007). Oros (2007) showed good to moderate water quality and moderate sediment quality with respect to heavy metal contamination, but the actual contamination level as well as the origin of the pollution was not approached.

In this regard, the authors of the current study aim to quantify the anthropogenic influences on the metal contamination level in Tasaul Lake by using some pollution indices. Furthermore, the authors use multivariate statistical techniques for identifying the sources of metal enrichment and highlighting the most polluted sites in the lake, thus providing valuable information for management decisions aiming at improving the quality of Tasaul ecosystem services.

## Materials and methods

### Study area

Tasaul Lake (ca. 20 km north of Constanta, the most important city of the Dobrudja region) is a maritime liman, formed as an extension of the Casimcea River valley and separated from the Black Sea by a coastal bar (Popescu and Caraivan 2002–2003). It has an elongated and slightly meandering shape owing to alternating promontories and relatively large gulfs (Breier 1976). Its shores are mostly high (3–12 m), composed of Jurassic limestone in the northern part and green schist in the southern part (Breier 1976).

**Fig. 1:**
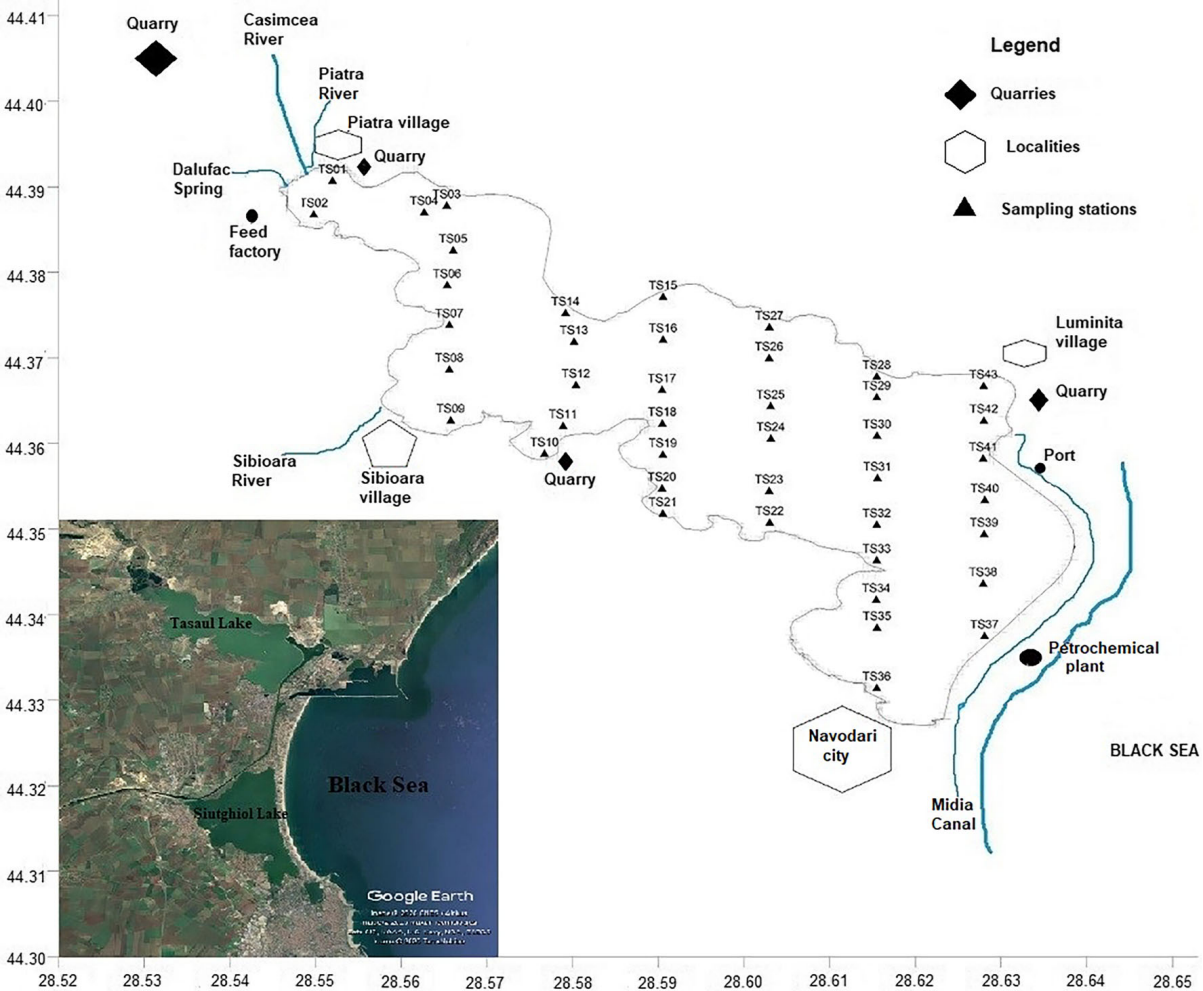
Location of the study area and sampling sites

The lake has an area of ca. 2025 ha (Cernisencu et al. 2007) and a mean depth of 2.4 m (maximum depth of 3.75 m), according to Breier (1976). There are two islands on the lake: Ada Island, with an area of 30.3 ha, and La Ostrov Island, with an area of 3 ha (Breier 1976) (Fig. 1).

South of the lake is Navodari (Fig. 1), an important city in the region due to industrial and, recently, touristic activities. The most important industrial facility is the petrochemical plant, to the east of the lake, a potential source of contaminants, mainly through its emissions. Other potential pollution sources linked to Navodari are domestic wastewater, waste disposal, and road traffic.

Apart from Navodari, there are three small villages around the lake: Piatra, located in the northwest, Sibioara, in the west, and Luminita, in the east (Fig. 1). The main human activities around these villages are mining and agriculture. Near each of the abovementioned villages, there are limestone (Piatra and Luminita) and greenschist (Sibioara) quarries, which can be seen as potential sources of metal pollution through quarry dust, which may harbor quite high levels of metals; leaching of metals from waste rock dumps; heavy car traffic, etc. (Tiimub et al. 2015). Other human activities carried out near these villages that might impact Tasaul Lake are animal husbandry (Sibioara), a feed factory (north of Sibioara), and port activities (Luminita port, located on the branch of the Poarta Alba—Midia Navodari Canal, which goes along the eastern lake border).

The northwestern part of the lake has been subject to hydrotechnical works aimed at developing fishing activities (there is a large fishing farm next to the mouth of the Casimcea River) (Alexandrov et al. 2008; Alexandrov and Bloesch 2009). The Casimcea River (mean flow of 0.22 m^3^/s), the main tributary of Tasaul Lake with a relatively large catchment area (830 km^2^) (Mateescu et al. 2007), has been subject to hydrotechnical works upstream, which modified the flow patterns and consequently the suspended solids, nutrients, and contaminants discharged into the lake (Mateescu et al. 2007). Other tributaries are two small rivers, the Piatra and the Sibioara, and the Dalufac spring (Mateescu et al. 2007), which enter the lake in northwest, south, and west, respectively (Fig. 1).

### Sampling and analytical procedures

The sampling program was carried out in September 2019 onboard the motorboat Carina. A total of 43 sediment samples were collected using a Van Veen grab 1000 cm^2^ (manufactured by KC Denmark). The 2-cm-deep surface sediment was sliced for each sediment sample. The subsamples were stored in plastic bags and kept at 0–4 °C until subsequent analyses.

The grain size of the sediments was determined with a Mastersizer 2000 laser diffraction granulometer (Mastersizer 2000E, ver. 5.20) and associated dispersion units (Malvern Instruments, UK) with a measurement precision of 1% and result reproducibility below 1%. The granulometric fractions are in accordance with the Udden-Wentworth dimensional scale with sand/silt and silt/clay boundaries of 63 μm and 4 μm, respectively. The Shepard ternary diagram was used for lithological classification of sediment samples (Sheppard 1954).

Before geochemical analyses, the sediments were oven dried (24–48 h/105 °C), ground, and homogenized with a mortar and pestle. The total organic carbon (TOC) concentrations were determined by the Wakley-Black titration method modified by Gaudette et al. (1974). The concentrations of Al, Cr, Cu, Ni, Zn, As, V, and Pb were measured by X-ray fluorescence spectrometry using an EDXRF Spectro Xepos spectrometer with a Pd/Co tube and XRF AnalyzerPro software (v. 3.3.2). A Direct Mercury Analyzer (DMA 80 Milestone, Italy) was used for the analysis of Hg in sediment samples. This instrument is based on drying sample and thermal decomposition, followed by electrothermal atomization of mercury. To validate the analytical methodology, a certified reference material (NCS DC 73022) was used. The measured and certified values of element/compound concentrations were compared (Table 1). All of the results obtained for this reference material were statistically similar to the certified values (*p* < 0.05), demonstrating the reliability of the methodology and the estimated concentrations. The relative standard deviations of the measured replicates were all within ± 5%.

**Table 1:** Measured and certified values of standard material NCS DC 73022

Element	Measured value (mg/kg) ± SD	Certified value (mg/kg) ± SD	Recovery (%)
Cr	75 ± 2.46	72 ± 3	104
Al_2_O_3_	12.88 ± 0.31	13.61 ± 0.12	94.6
As	289 ± 2	**304 ± 20**	**95**
Ni	32.2 ± 0.12	29 ± 1	110
Cu	503 ± 4.58	483 ± 20	104
Pb	140.3 ± 1.53	126 ± 5	111
V	100.1 ± 0.81	101 ± 3	99
Zn	861 ± 11.79	874 ± 19	98.5
Hg	0.122 ± 0.005	0.115 ± 0.023	106

### Pollution indices

The level of sediment pollution in Tasaul Lake was assessed based on several indices, i.e., the enrichment factor (EF), contamination factor (CF), and pollution load index (PLI).

EF is widely used to separate natural variations in metals of from the metal fraction that is associated with sediments due to anthropogenic activities. The EF for each element was calculated to assess the actual contamination level (Sakan et al. 2009) using the following formula:

$$ \mathrm{EF}=\mathrm{E}{\mathrm{F}}_i=\frac{C_{i,s}}{C_{i,b}}\frac{c_{\mathrm{ref},b}}{c_{\mathrm{ref},s}} $$

EF values were interpreted as suggested by Acevedo-Figueroa et al. (2006), where EF < 1 indicates no enrichment, < 3 is minor, 3–5 is moderate, 5–10 is moderately severe, 10–25 is severe, 25–50 is very severe, and > 50 is extremely severe.

CF is the ratio obtained by dividing the concentration of each metal in the sediment by the background value (concentration in uncontaminated sediment), according to the following formula:

$$ \mathrm{CF}=\mathrm{C}{\mathrm{F}}_i=\frac{C_{i,s}}{C_{i,b}} $$

CF values were interpreted as suggested by Hakanson (1980), where CF < 1 indicates low contamination, 1 < CF < 3 is moderate contamination, 3 < CF < 6 is considerable contamination, and CF > 6 is very high contamination.

For the entire studied area, PLI was determined as the *n*th root of the product of n CFs, according to the formula below:

$$ \mathrm{PLI}=\sqrt[n]{\Big(\mathrm{CF}1\times \mathrm{CF}2\times \mathrm{CF}3\times \dots .\times \mathrm{CF}\mathrm{n}} $$

This empirical index provides a simple, comparative means for assessing the level of heavy metal pollution. PLI > 1 suggests that pollution exists; PLI < 1 means that there is no metal pollution (Tomlinson et al. 1980).

*C*_*i,s*_ is the concentration of metal *i* in the sample, and *c*_*i,b*_ is the background concentration of metal *i*. Since no background data for metals in uncontaminated sediments in the study area are available, the concentration of metal *i*, *C*_*i,b*_ (mg/kg dry matter), in the surface sediments of upper continental crust (UCC) reported by Rudnic and Gao (2003) was used as a background value (i.e., c_*Al,b*_ = 81,500 mg/kg, *c*_*As,b*_ = 4.8 mg/kg, *c*_*Pb,b*_ = 17 mg/kg, *c*_*Cu,b*_ = 28 mg/kg, *c*_*Hg,b*_ = 0.05 mg/kg, *c*_*Ni,b*_ = 47 mg/kg, *c*_*Cr,b*_ = 92 mg/kg, *c*_*V,b*_ = 97 mg/kg, and *c*_*Zn,b*_ = 67 mg/kg). C_*ref*_,_*s*_ and C_*ref,b*_ are the concentrations of reference elements in the samples and in surface sediments of UCC (Rudnic and Gao 2003). In this study, Al was chosen to normalize the elements due to its high natural abundance in the UCC. It is one of the most conservative metals, and is not affected by anthropogenic activities (Schropp et al. 1990; Liaghati et al. 2003).

### Data processing

xlSTAT 7.5.2. Software (AddinSoft 2020) was used for statistical analyses. Multivariate statistical techniques, which included Pearson correlation and principal component analysis (PCA), were applied to investigate the potential sources of heavy metals in the sediments. Correlation analysis presents a statistical characterization of a quantitative variable by other quantitative variables to depict their relationships using the correlation coefficient (Pearson). PCA was performed on the metal concentration in sediment samples to further understand the grouping of heavy metals from the same source. The validity of PCA was evaluated by applying the Kaiser-Meyr-Olkin test and Bartlett’s test. The principal components (PCs) with eigenvalues greater than one were considered to be relevant (Yang et al. 2014). To consider the contribution of elements to a given group, the components with factor loadings of > 0.6, 0.4–0.6, and 0.3–0.4 were classified as highly, moderately, or weakly associated with elements in that class, respectively. Similar classification procedures have been used in similar studies focusing on the identification of heavy metal sources in sediments (Wu et al. 2014; Javed et al. 2018; Maina et al. 2019).

Agglomerative hierarchical clustering (AHC) analyses were carried out by using Ward’s method (Ward 1963), with the Euclidean distance (proximity matrix) as a measure of dissimilarity to assess the associations among the sampling sites with respect to the pollution indices. AHC is an iterative classification method that initiates clustering by calculating the dissimilarity between different groups of objects and then clusters the objects together to minimize a given agglomeration criterion (Benson et al. 2016).

Surfer® (Golden Software, LLC) was used to map the spatial distribution of variables (grain size, TOC, heavy metals, and PLI) in the study area.

## Results and discussion

### Particle size and TOC

**Fig. 2:**
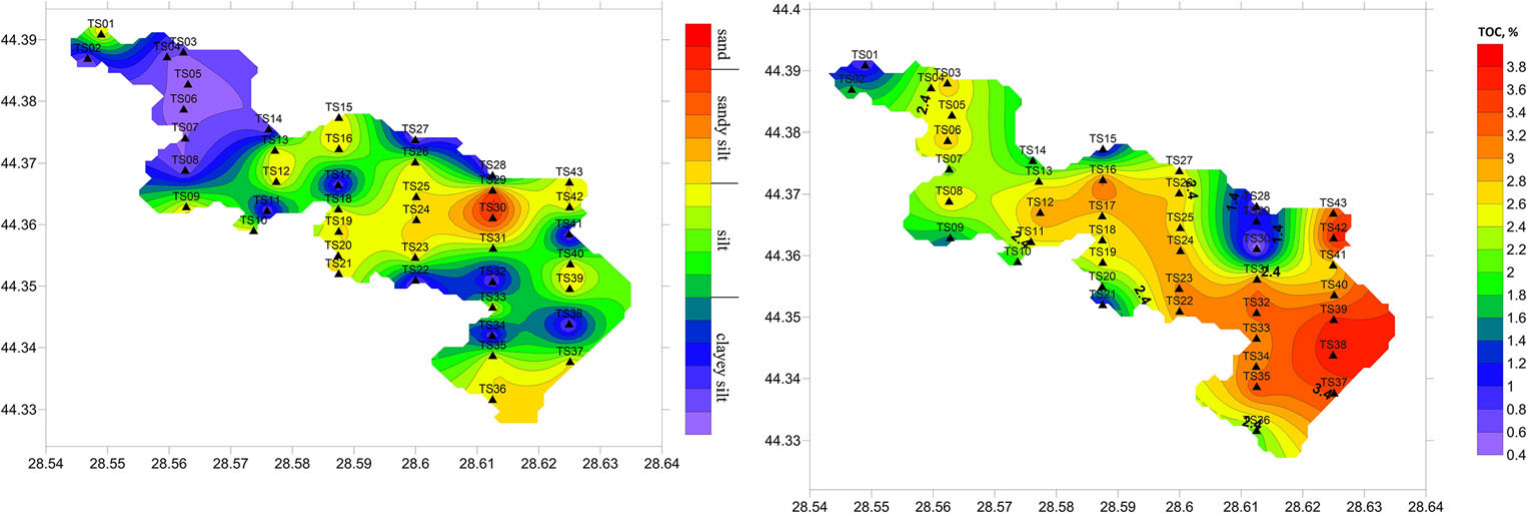
Grain size and TOC in the surface sediments of Tasaul Lake

Generally, the study area is covered by clayey silt (40% of samples), mostly in the western part, and sandy silt (56% of samples), except for two sampling stations located in the vicinity of Ada Island (TS29 and TS30), where the coarse fraction (sand + gravel) represents > 80% (Fig. 2). The silt fraction varied from 9.2% (station TS29) to 79.9% (stations TS01 and TS02), with an average of 69.7 ± 14.1%. The highest percentages of clay (> 20%) were found in the western part of the study area (stations TS02, TS03, TS04, TS05, TS06, and TS11), where the main tributary, the Casimcea River, enters the lake, while the lowest percentage was determined at station TS29 (1.15%).

The TOC concentrations in the surface sediments fluctuated from 0.29 to 3.73%, with an average of 2.74 ± 0.86% (Table 2). The lowest TOC was recorded near Ada Island (station TS30), while the highest TOC was measured in the eastern part of the lake (maximum at station TS38) (Fig. 2). However, the TOC concentrations were relatively high, with values > 2% being measured in 63% of samples. The TOC values showed significant negative correlations with sand and gravel fractions and positive correlations with silt and clay fractions (Table 3).

**Table 2:** Descriptive statistics for the studied variables (TOC, Al, and heavy metals)

	TOC (%)	Al (mg/kg)	Cr (mg/kg)	Ni (mg/kg)	Cu (mg/kg)	Zn (mg/kg)	As (mg/kg)	V (mg/kg)	Pb (mg/kg)	Hg (mg/kg)
Min.	0.29	41,506	72.3	27.74	15.12	46.4	5.86	54.40	16.50	0.02
Max	3.73	67,871	103.0	57.60	83.50	133.0	17.70	95.90	34.46	0.14
Mean	2.47	49,772	83.70	42.53	34.27	84.40	12.49	76.45	26.30	0.06
SD	0.86	4529	7.0	6.45	11.18	16.1	2.99	9.06	4.71	0.02
CV (%)	34.8	9.1	8.3	15.2	32.6	19.1	23.9	11.8	17.9	38.4

**Table 3:** Correlation matrix (Pearson) for the particle size, TOC, and heavy metals

Location	Element mg/kg								Reference
	Cu (mg/kg)	Zn (mg/kg)	Hg (mg/kg)	As (mg/kg)	Ni (mg/kg)	Cr (mg/kg)	V (mg/kg)	Pb (mg/kg)	
Tașaul Lake, Romania	15.1–83.5	46.4–133	0.02–0.14	5.9–17.7	27.7–57.6	72.3–103	54.4–95.9	16.5–34.5	Current study
Tasaul Lake,Romania	18.6–57.4	-	-	-	10.7–92.1	-	-	26.9–126	Oros (2007)
Tabacarie Lake, Romania	44–380	307–894	-	-	41–76	85–128	42–93	44–376	Caraivan et al. (2011)
Siutghiol Lake, Romania	15–40.6	> 1000	0.02–0.28			5.0–82.0		> 100	Bucur Arpenti et al. (2014)
Lesina Lake, Italy	7.56–65.15	9.45–62.01	-	0–26.12	4.78–41.04	9.44–64.74	12.22–91.08	6.60–76.35	Spagnoli and Andresini (2018)
Gardno Lake, Poland	0.7–20.3	7.5–113.4	-	-	-	-	-	7.4–61.6	Trojanowski et al. (2007)
San Puoto Lake, Italy^a^	41	67	-	18	28	54	66	20	Alvisi and Dinelli (2002)
Akkulam-Veli, India	4.6–92.8	12.2–209.0			11.4–69.0	16.8–83.2		8.4–78.4	Sheela et al. (2012)
Burrulus Lake, Egypt^a^	23.6	75.1				53.9		22.8	El-Amier et al. (2017)

^a^Results are expressed as means

### Metal concentrations and spatial distribution

The mean values of metal concentrations showed a decreasing order: Al >>> Zn > Cr > V>Ni > Cu > Pb > As > Hg. The minima, maxima, means, standard deviations, and coefficients of variation (CV, %) for each studied metal and TOC (*n* = 43) are presented in Table 2.

**Fig. 3:**
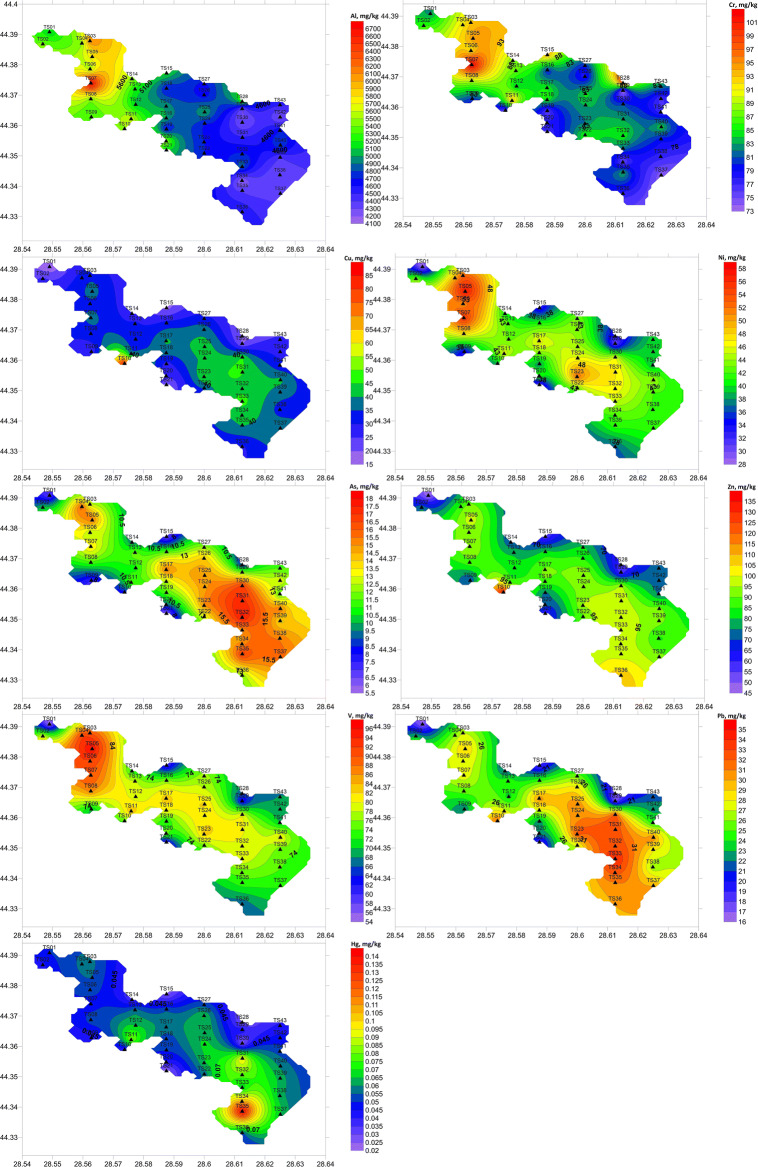
Metal distribution in the surface sediments of Tasaul Lake

Metal concentrations in the surface sediments presented low to moderate spatial variability, with CVs ranging from 8.3 to 38.4% (minimum for Cr and maximum for Hg) (Table 2). Al, Cr, Ni, and V showed quite similar distributions, with the highest concentrations in the northern part of the lake (Al and Cr at station TS07 and Ni and V at station TS05) (Fig. 3). Cu and Zn showed maxima at station TS10, while the highest concentrations of As, Pb, and Hg were measured in the southeastern part of the lake (stations TS32, TS33, and TS35, respectively) (Fig. 3). The lowest concentrations were found at stations TS01 (Ni, Cu, Zn, V, and Pb), TS21 (As and Hg), TS29 (Cr), and TS34 (Al) (Fig. 3).

Romanian coastal lakes, particularly Tasaul Lake, have been quite poorly investigated in terms of sediment quality (Oros 2007; Bloesch and Alexandrov 2007); thus, it is quite difficult to assess the temporal variability in heavy metals in sediments. However, by comparing our data with those collected by Oros (2007) in Tasaul Lake (the only available study), it can be remarked that quite similar Cu concentrations and significantly lower Pb and Ni levels were measured in 2019. Regarding the spatial distribution, the current data showed similar patterns of Ni and Pb for both periods (higher Ni concentrations in the west of lake and higher Pb concentrations in east of the lake). Cu showed some differences in its spatial distribution; in 2019, higher concentrations were measured in the west, contrary to 2005–2007, when higher concentrations were observed in the east.

The heavy metal concentrations measured in this study were also compared with data collected from two other shallow coastal lakes (Tabacarie and Siutghiol), also located in the Constanta area, south of Tasaul Lake, as well as some coastal lakes from other countries (Table 4).

**Table 4:** Comparative heavy metal concentrations (mg/kg) in the sediments of some coastal lakes

	Sand + gravel (%)	Silt (%)	Clay (%)	TOC (%)	Al (mg/kg)	Cr (mg/kg)	Ni (mg/kg)	Cu (mg/kg)	Zn (mg/kg)	As (mg/kg)	V (mg/kg)	Pb (mg/kg)	Hg (mg/kg)
Sand + gravel	*1*												
Silt	*− 0.978*	*1*											
Clay	*− 0.801*	*0.657*	*1*										
TOC	*− 0.443*	*0.443*	*0.327*	*1*									
Al	*− 0.388*	*0.310*	*0.506*	*− 0.308*	*1*								
Cr	*− 0.359*	0.268	*0.526*	**−** 0.188	*0.752*	*1*							
Ni	*− 0.304*	0.187	*0.558*	*0.327*	*0.400*	*0.481*	*1*						
Cu	*− 0.016*	**−** 0.006	0.073	*0.430*	**−** 0.011	**−** 0.133	*0.442*	*1*					
Zn	**−** 0.137	0.081	0.262	*0.447*	0.035	**−** 0.020	*0.606*	*0.892*	*1*				
As	0.072	**−** 0.134	0.124	*0.592*	*− 0.317*	**−** 0.032	*0.641*	*0.446*	*0.583*	*1*			
V	*− 0.312*	0.182	*0.602*	*0.365*	*0.470*	*0.500*	*0.953*	*0.445*	*0.591*	*0.558*	*1*		
Pb	**−** 0.088	0.035	0.217	*0.498*	**−** 0.118	**−** 0.042	*0.653*	*0.810*	*0.905*	*0.770*	*0.605*	*1*	
Hg	**−** 0.270	0.255	0.243	*0.615*	**−** 0.272	**−** 0.089	0.272	*0.314*	*0.406*	*0.599*	0.229	*0.537*	*1*

Values in italics are significant at a confidence level *α* = 0.05

Data reported by Caraivan et al. (2011) and Bucur Arpenti et al. (2014) for Tabacarie and Siutghiol Lakes showed much higher concentrations for Cu, Ni, Zn, and Pb (Tabacarie Lake) and Hg, Zn, and Pb (Siutghiol Lake), respectively (Table 4). These findings are in line with the pressures exerted by recreational activities, tourism, untreated/poorly treated domestic wastewater, and urban extension (Siutghiol), which are much stronger at these other lakes than in Tasaul Lake.

The heavy metal concentrations measured in other coastal lakes generally did not show significant differences compared with those found in the current study in Tasaul Lake (Table 4). Generally, the results show that the heavy metal concentrations in the study area fall within the range of those of other regions listed in the Table 4.

### Heavy metal contamination sources

Potential sources of the measured heavy metals were identified by using Pearson correlations and PCA. Pearson’s correlation coefficients for metals, TOC, and grain size in the surface sediments of Tasaul Lake are presented in Table 3.

TOC and particle size are two important factors in the distribution of metals in sediments (Aloupi and Angelidis 2001; Dou et al. 2013). The correlation matrix (Table 3) showed significant positive relationships between TOC and all heavy metals, except for Cr, suggesting the major role played by organic matter in the transport processes of these metals (Kükrer et al. 2020). The significant positive correlations among Cu, Zn, As, Pb, and Hg might indicate that they originate from the same sources and have similar transformation and migration processes (Wang et al. 2012). Ni and V showed a significant positive correlation with each other but also with Cu, Zn As, and Pb (Table 3).

Generally, the heavy metal concentrations in the surface sediments increased with decreasing grain size (O’Reilly-Wiese et al. 1995). Nevertheless, among the heavy metals, only Cr, V, and Ni showed a significant positive correlation with the clay fraction, which might suggest lithogenic sources for those metals (Dou et al. 2013). As a major component of clayey minerals, Al was positively correlated with the clay fraction, but it showed significant positive correlations with Cr, Ni, and V as well, thus suggesting a natural origin for these heavy metals.

The PCA results showed three principal components with eigenvalues > 1, accounting for approximately 85% of the total variance (Table 5). The first principal component (PC1), accounting for 46.9% of the total variance, was categorized by high positive loadings of TOC (0.649), Ni (0.819), Cu (0.757), Zn (0.880), As (0.823), V (0.781), and Pb (0.931). The abovementioned heavy metals represent the pollution group and are significantly associated with each other. The positive loading of Hg (0.598) was very close to 0.6; thus, this metal might be considered to be significantly associated with the abovementioned elements. The high loading factors indicated mainly anthropogenic sources of pollution.

**Table 5:** Factor loadings, eigenvalues, and variance in the PCA matrix of heavy metals in the surface sediments of Tasaul Lake

Parameters	PC1	PC2	PC3
TOC (%)	0.649	− 0.366	− 0.420
Al (mg/kg)	0.017	0.929	0.103
Cr (mg/kg)	0.110	0.877	− 0.252
Ni (mg/kg)	0.819	0.475	− 0.161
Cu (mg/kg)	0.757	− 0.118	0.583
Zn (mg/kg)	0.880	− 0.046	0.407
As (mg/kg)	0.823	− 0.233	− 0.281
V (mg/kg)	0.781	0.535	− 0.108
Pb (mg/kg)	0.931	− 0.143	0.211
Hg (mg/kg)	0.598	− 0.381	− 0.418
Eigenvalue	4.963	2.515	1.092
Variance (%)	49.63	25.15	10.92

PC2 accounted for 25.1% of the total variance (Table 5) and was dominated by Al and Cr, with high positive loadings (0.929 and 0.877, respectively), thus suggesting the natural origin for those two metals. Moderate positive loadings were also found for Ni (0.435) and V (0.575), which might indicate that those metals originated from both natural (PC2) and anthropogenic sources (PC1).

PC3 accounted for 10.92% of the total variance and exhibited moderate positive loadings for Cu (0.583) and Zn (0.407) and moderate negative loadings for TOC (− 0.420) and Hg (− 0.418) (Table 5). This result might indicate different pollution sources for Cu and Zn other than the common sources for most heavy metals determined from PC1.

### Heavy metal contamination of surface sediments (pollution indices)

**Fig. 4:**
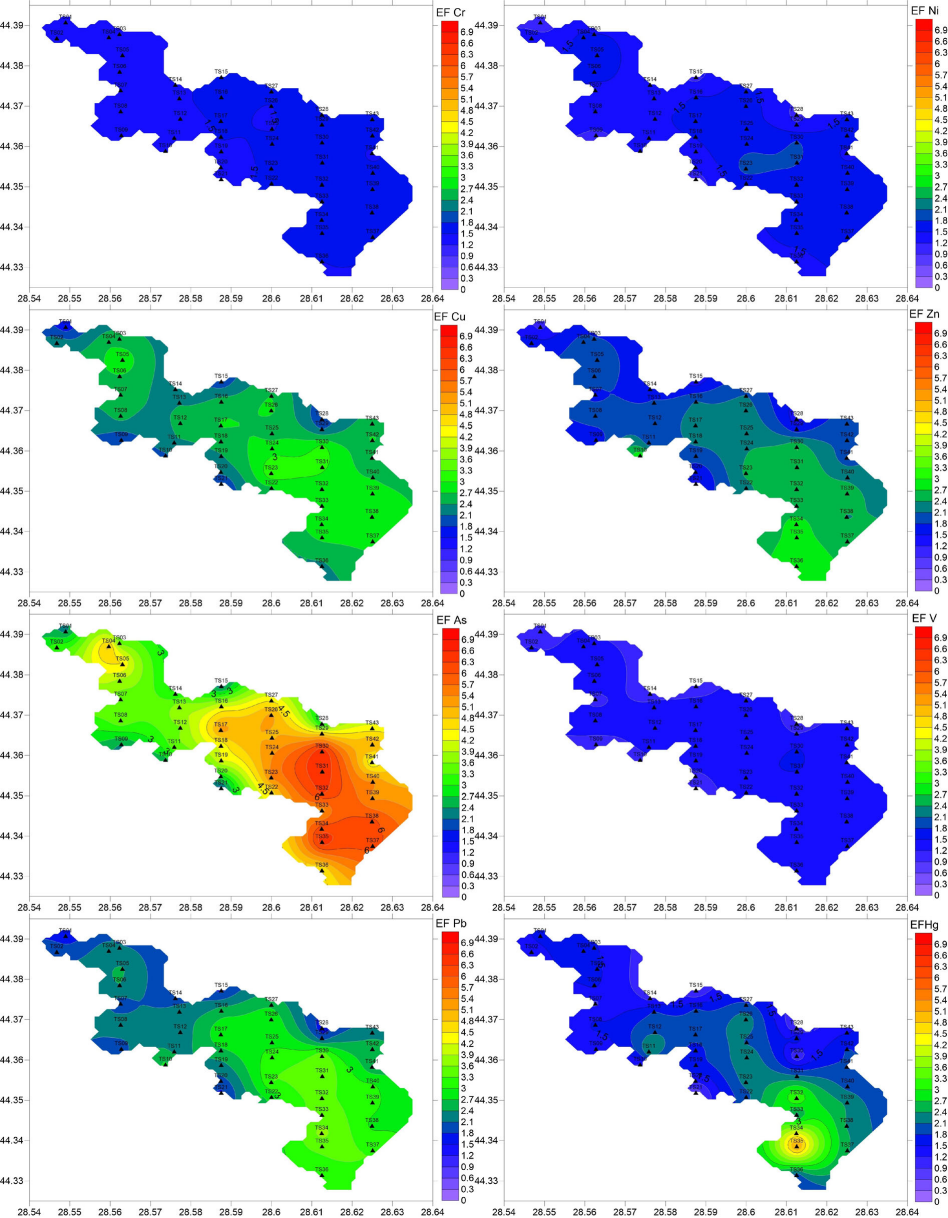
Spatial distribution of EFs in Tasaul Lake

EF showed values ranging from 1.2–1.7 (Cr), 1.0–1.9 (Ni), 0.9–4.4 (Cu), 1.1–3.0 (Zn), 2.0–6.6 (As), 0.9–1.6 (V), 1.6–3.7 (Pb), and 0.7–5.3 (Hg). Arsenic showed the highest mean EF (4.4 ± 1.3), with ca. 35% of values between 5 and 10 (most of them in the south of the lake) (Fig. 4), thus suggesting moderate to severe enrichment of As. The second largest mean was found for Pb (2.6 ± 0.6), with 25% of the EF values (mostly in the south of the lake) ranging from 3 to 5 (Fig. 4), suggesting low to moderate enrichment. The other heavy metals generally presented EF values from 1– to 3 (Fig. 4), with means of 2.0 ± 0.7 (Cu), 2.1 ± 0.5 (Zn), 1.9 ± 0.9 (Hg), 1.5 ± 0.1 (Cr), 1.5 ± 0.2 (Ni), and 1.3 ± 0.2 (V), suggesting minor enrichment in the study area.

**Fig. 5:**
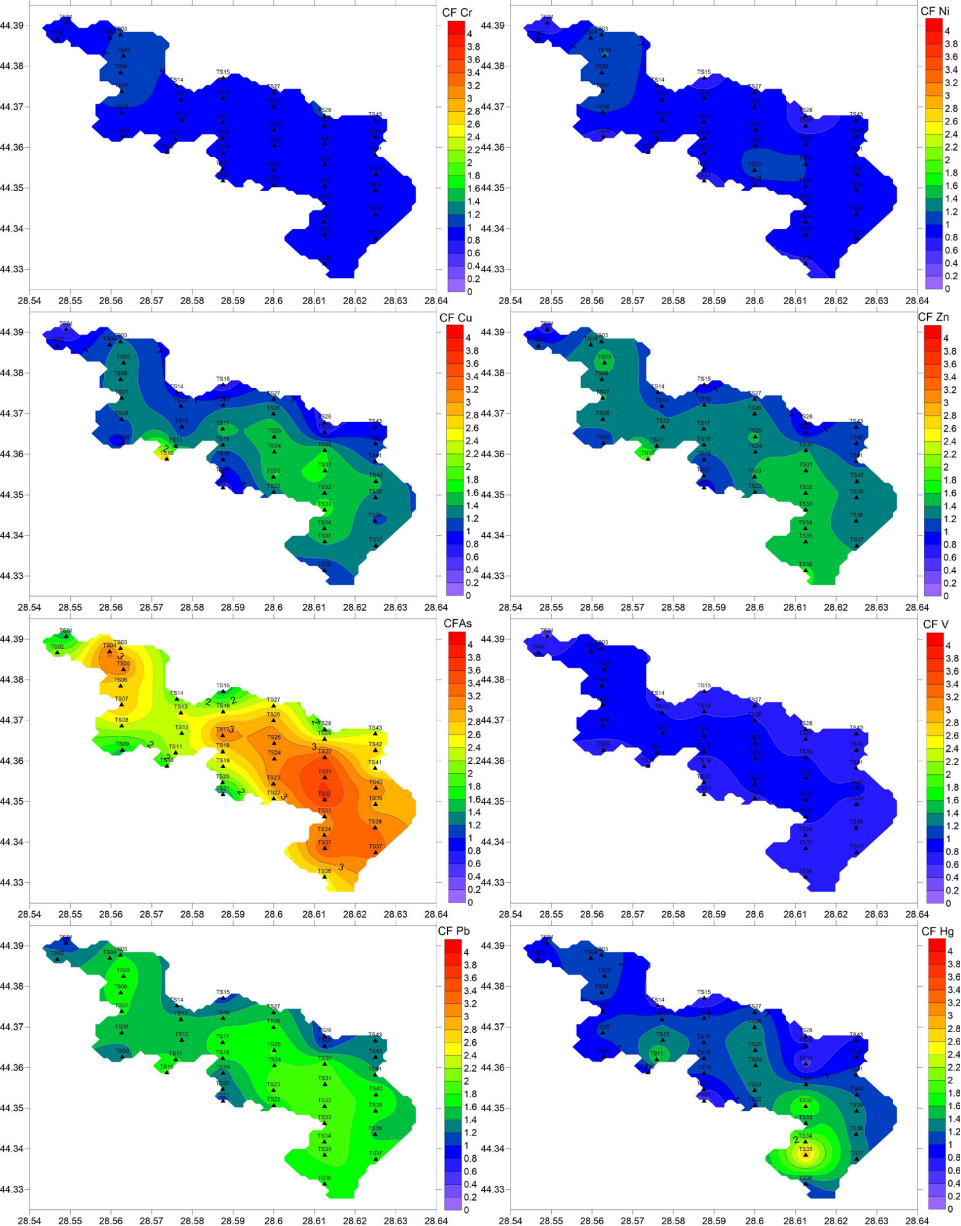
Spatial distribution of CFs in Tasaul Lake

CF was also used to assess metal contamination in Tasaul Lake sediments. The CF values were from 0.8 to 1.1 (Cr), 0.6–1.2 (Ni), 0.6–3.0 (Cu), 0.7–2.0 (Zn); 1.2–3.7 (As), 0.6–1.0 (V), 1.0–2.0 (Pb), and 0.4–2.9 (Hg). Similar to EF, arsenic showed the highest mean (2.6 ± 0.6), with more than 30% of values (the southern part of the lake) between 3 and 6 (Fig. 5), indicating moderate to considerable contamination. Cu, Zn, Pb, and Hg presented means between 1 and 3 (1.2 ± 0.4, 1.3 ± 0.2, 1.6 ± 0.3, and 1.2 ± 0.2, respectively) (Fig. 5), corresponding to moderate contamination. V, Cr, and Ni exhibited the lowest CF means (0.8 ± 0.1, 0.91 ± 0.08, and 0.93 ± 0.1, respectively) (Fig. 5), suggesting no contamination.

The CF and EF values highlight the relatively high level of anthropogenic arsenic in the surface sediments (Figs. 4 and 5). Some authors explained that a higher level of As might originate either from excessive use of agrochemicals and fertilizers (Yisa et al. 2011; Bai et al. 2012; Cai et al. 2015; Villalobos-Castaneda et al. 2016; Javed et al. 2018) or from atmospheric inputs such as the precipitation of aerosol particles released by quarrying activities (Tiimub et al. 2015). The multivariate analysis results, particularly the strong relationship with TOC, suggest a terrestrial source; thus, the high arsenic contamination of surface sediments might be attributed mainly to agricultural runoff.

The second-ranked heavy metal in terms of pollution level in Tasaul Lake sediments was Pb. According to the PCA and correlation matrix, Pb contamination was primarily attributed to anthropogenic sources, such as leaded gasoline, municipal runoff, and atmospheric deposition (Mukai et al. 1994; Sheela et al. 2012; Mohiuddin et al. 2012). Higher CF and EF values were found in the southeastern part of the lake, which is more strongly impacted by the untreated/poorly treated domestic discharges (from the Navodari city area), urban runoff, vehicle exhaust (car traffic is more intense in Navodari and the surrounding area), and most likely emissions released by the petrochemical plant (Cai et al. 2015; Cao et al. 2020). However, the significant positive correlation between TOC and Pb (Table 3) suggests that untreated/poorly treated wastewater and urban runoff are the main pollution sources. The considerable reduction in the use of leaded petrol has contributed to the decrease in the atmospheric input of Pb (Islam et al. 2015; Kuriata-Potasznik et al. 2016).

The strong relationships between Cu, Zn, and Pb (Table 3) suggest quite similar pollution sources. Cu and Zn might originate from agricultural runoff and industrial and domestic wastewater as terrestrial sources (Njogu et al. 2011; Sheela et al. 2012; Zeng and Wu 2013; Villalobos-Castaneda et al. 2016; Spagnoli and Andresini 2018), as well as quarrying activities (Etim and Adie 2012; Tiimub et al. 2015). The highest CFs and EFs for Cu (3.0 and 4.4, respectively) and Zn (2.0 and 3.0, respectively) were found at station TS10 in the southwest of the lake (Figs. 4 and 5). It is assumed that the pollution at this site is mainly related to quarrying activities (Etim and Adie 2012), but also to agricultural runoff, as Cu and Zn are used in fungicides and fertilizers (Momtaz 2002). However, Zn also showed relatively high pollution indices (CF = 1.5–1.6, EF = 2.8–2.9) also in the southeast, in the proximity of the Navodari area (mainly due to urban runoff), while Cu showed higher values either in the south or in the east of the lake (Figs. 4 and 5), most likely due to agricultural runoff and domestic wastewater discharges and municipal runoff.

Mercury might enter the lake via untreated/poorly treated domestic wastewater discharges (Cai et al. 2015), through the precipitation of aerosol particles released from petrochemical industrial activities (Atoufi and Lampert 2002; Shakhova et al. 2016) and/or vehicle exhaust (Cai et al. 2015). Considering the very strong relationship with TOC (Table 3), the main source of Hg in the lake seems to be wastewater discharge. The spatial distribution of EFs and CFs (Figs. 4 and 5) showed the highest Hg pollution in the proximity of Navodari city (southeast of the lake) and seems to be related to the expansion of urbanization in the area and the poor domestic wastewater treatment system. However, the CFs and EFs indicate that the overall level of Hg pollution in Tasaul Lake sediments is relatively low (Figs. 4 and 5).

The lowest EFs and CFs were found for Cr, Ni, and V. Their spatial distribution, with slightly higher values in the west of the lake (Figs. 4 and 5), might suggest the weathering of greenschist from the Casimcea River watershed, as well as quarry dust resulting from the exploitation of the northwestern quarry, as the main sources. Nickel presented slightly higher CF (1.1) and EF (1.9) values also at stations TS23 and TS30, most likely in connection with agricultural runoff and quarrying activities in northeastern part of the lake.

**Fig. 6:**
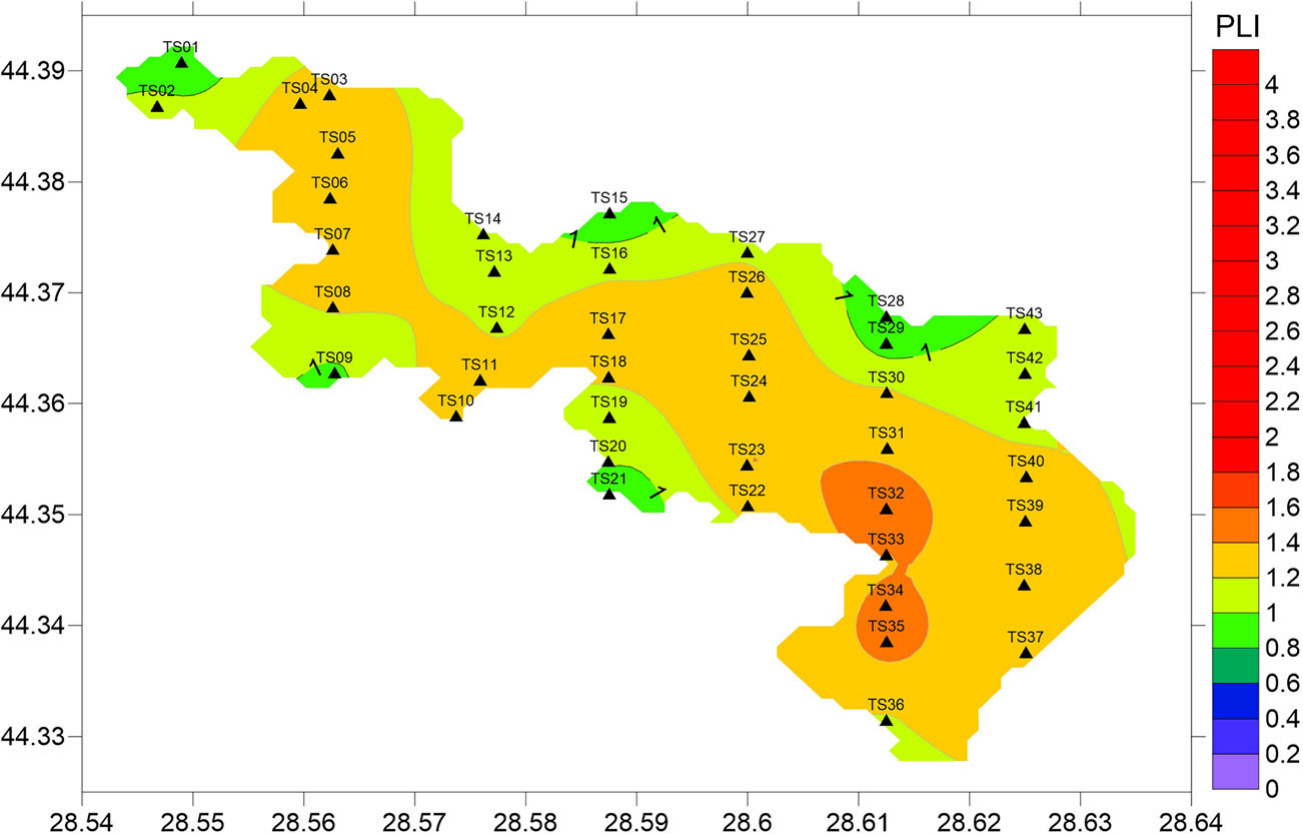
Spatial distribution of PLI in Tasaul Lake

PLI was used to assess the spatial variability in pollution intensity. Its values ranged from 0.8 to 1.5, more than 85% of the values being > 1, suggesting that heavy metal pollution exists in the surface sediments of Tasaul Lake. The greatest PLIs were found in the south and southeast of the lake (stations TS35, TS34, TS33, and TS32) (Fig. 6), most likely due to the proximity to Navodari city and the petrochemical plant, as well as agricultural activities, while the smallest PLI was measured in one of the gulfs of the eastern part of the lake (station TS21). High PLIs were also observed in the west of the lake (stations TS03, TS05, and TS07) (Fig. 6), which might be related to discharges from the Casimcea and the small river watersheds, as well as quarrying activities.

**Fig. 7:**
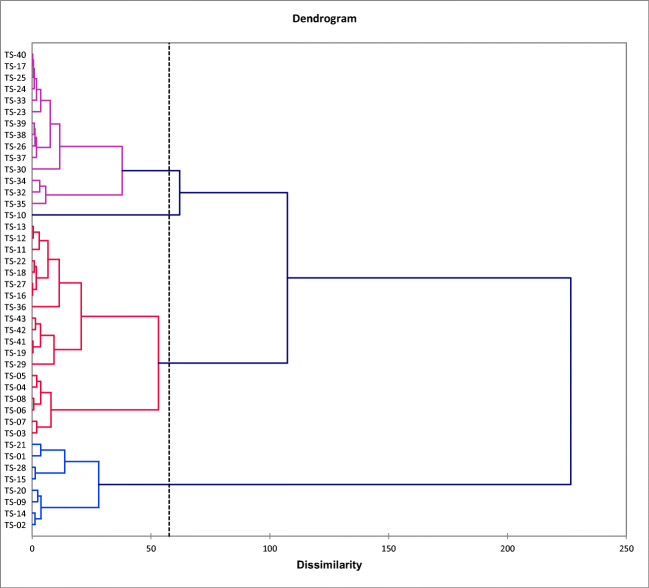
Dendrogram showing clustering of sampling stations

Hierarchical cluster analysis was applied to datasets of pollution indices (CFs and EFs) to group similar sampling sites (spatial variability). The HCA results were rendered as a dendrogram (Fig. 7), where all the sampling stations were clustered into four statistically significant groups.

The first group consists of stations generally located very close to the lake banks (TS01, TS02, TS09, TS14, TS15, TS20, TS21, and TS28) and showed the lowest PLIs (from 0.8 to 1.0, with an average value of 0.89). Those stations are characterized either by relatively high percentages of sand fraction (> 15%) or lower TOC (< 1%), thus explaining the low accumulation of heavy metals in sediments.

The second group includes 19 stations (PLIs from 0.9 to 1.4, with an average of 1.19) located mostly in the western part of the lake. The highest PLIs within this group were observed at the stations located in the area of influence of the lake’s tributaries (stations TS03, TS04, TS05, TS06, TS07, and TS08). These stations are characterized by higher percentages of clay (> 20%) and TOC (> 2%), explaining the higher accumulation of heavy metals in sediments. The group also includes the stations located in the northeastern edge of the lake (TS41, TS42, and TS43), very close to the Luminita quarry.

Generally, the second group is impacted by agricultural runoff from the surrounding fields, the Casimcea River and other small temporary river discharges (including agricultural runoff within their watersheds and domestic wastewater discharges), as well as quarrying activities (especially in the northeast of the lake).

The third group consists only of one station, TS10, with a relatively high PLI (1.29), mainly owing to high Cu, Zn, and Pb concentrations.

The fourth group contains 14 stations located mostly in the eastern and southeastern parts of the lake and shows a mean PLI of 1.35. Generally, those stations are impacted by agricultural runoff, discharges of domestic wastewater from Navodari city and the surrounding area, industrial activity (especially from the petrochemical plant), road traffic, urbanization, etc. The highest contributions to the PLI values come from As, Pb, Hg, Cu, and Zn.

## Conclusions

Grain size, Al, TOC, and heavy metals (Cr, Ni, Cu, Zn, As, V, Pb, and Hg) were measured in the surface sediments of Tasaul Lake. The mean concentrations of heavy metals were 83.70 mg/kg (Cr), 42.53 mg/kg (Ni), 34.27 mg/kg (Cu), 84.40 mg/kg (Zn), 12.49 mg/kg (As), 76.45 mg/kg (V), 26.30 mg/kg (Pb), and 0.06 mg/kg (Hg). As, Pb, and Hg exhibited the highest concentrations in the south and southeast of the lake (mostly affected by urbanization and industry). Cu and Zn showed maxima in the southwest of the lake, while Cr, Ni, and V showed maxima in the western part.

According to the multivariate statistical analyses, Cr predominantly originated from lithogenic components naturally weathered from the surrounding land, while As, Cu, Zn, Pb, and Hg contamination was related to human activities. Ni and V showed both lithogenic and anthropogenic sources. The most important anthropogenic sources were fertilizers and pesticides from agricultural runoff (As, Cu, Zn, and Pb), industrial and domestic sewage (Cu, Zn, Pb, and Hg), and quarry dust (Cu, Zn, Ni, and V).

The heavy metal pollution in Tasaul Lake was investigated based on the following indices: EF (enrichment factor), CF (contamination factor), and PLI (pollution index load). The results indicate that the enrichment of metals in surface sediments is dominated by As, followed by Pb. The other investigated heavy metals showed minor enrichment. The highest CF was also determined for As (moderate to considerable contamination), followed by Pb, Cu, and Zn (moderate contamination), while the lowest CFs, suggesting no contamination, were observed for Ni, Cr, and V.

Most PLI values were greater than 1, suggesting that pollution exists in the surface sediments of Tasaul Lake. According to the PLI spatial mapping and HCA analyses, the most polluted sites in the lake were in the east and southeast of the lake (due to urbanization, the petrochemical industry and agriculture), as well as in the western part, which is impacted by the tributary discharges and quarrying activities.

## Data Availability

The datasets generated during and/or analyzed during the current study are available from the corresponding author (DV) on reasonable request.
